# Investigating the experience of receiving podiatry care in a tertiary care hospital clinic for people with diabetes related foot ulcers

**DOI:** 10.1186/s13047-022-00556-1

**Published:** 2022-07-01

**Authors:** Emilee Kim Ming Ong, Caroline Fryer, Kristin Graham, Ryan Scott Causby

**Affiliations:** Allied Health and Human Performance, University of South Australia, Adelaide, South Australia 5001 Australia

**Keywords:** Diabetes related foot ulcers, Diabetes, Patient experience, Thematic analysis, Qualitative descriptive study

## Abstract

**Background:**

Diabetes related foot ulcers can have physical, social, emotional, and financial impacts on the daily life and wellbeing of many people living with diabetes. Effective treatment of diabetes related foot ulcers requires a multi-faceted, multi-disciplinary approach involving a podiatrist, other healthcare professionals, and the person with diabetes however, limited research has been conducted on the lived experience of podiatric treatment for diabetes related foot ulcers to understand how people are engaged in their ulcer management. Therefore, this study aimed to explore the lived experience of receiving podiatric treatment for diabetes related foot ulcers in a tertiary care outpatient setting.

**Methods:**

Ten participants were interviewed. All were male, with mean age of 69 (SD 15) years and currently undergoing podiatric treatment for a diabetes related foot ulcer in a tertiary care setting. Participants with diabetes related foot ulcers were purposively recruited from the outpatient podiatry clinic at a tertiary hospital in a metropolitan region of South Australia. Semi-structured interviews were conducted to gain insight into the lived experience of people receiving podiatric treatment for their foot ulcer and understand how this experience impacts their regular lifestyle. Data were analysed using a thematic analysis method.

**Results:**

Four themes were identified that add an understanding of the lived experience of participants: ‘Trusting the podiatrists with the right expertise’, ‘Personalised care’, ‘Happy with the service, but not always with prescribed care’, and ‘It’s a long journey’. Participants described professional behaviour including high organisation and hygiene practices and demonstrated expertise as key factors influencing their trust of a podiatrist’s care. Information tailored to individual needs was helpful for participants. Offloading devices and ulcer dressings were often burdensome. Podiatric treatment for foot ulcers was a lengthy and onerous experience for all participants.

**Conclusions:**

The findings of this study suggest podiatrists can use practical strategies of maintaining consistency in who provides podiatry care for the person, demonstration of high organisational and hygiene standards, and using innovation to adapt information and equipment to suit an individual’s lifestyle to support positive experiences of podiatric ulcer care. There is a need for further research to understand how a person’s experience of podiatric ulcer care differs amongst genders, cultural groups, and healthcare settings to facilitate positive care experiences and reduce treatment burden for all people with diabetes requiring podiatric ulcer treatment.

**Supplementary Information:**

The online version contains supplementary material available at 10.1186/s13047-022-00556-1.

## Background

Diabetes related foot ulcers (DFUs) have significant physical, social, economic, and psychological impacts for many people living with diabetes. A DFU is a full thickness wound in the epithelium that extends into or through the dermis layer and may cause damage to underlying structures including nerves and tissues distal to the ankles [1, 2]. Twenty-five percent of people living with diabetes will develop a DFU within their lifetime [3]. Male gender and neuropathy are key predictors for the development of DFUs [4]. The high prevalence of DFUs presents a significant burden on individuals, communities, and healthcare systems around the world.

Poorly managed DFUs can compromise the viability of an entire lower limb. Infection, gangrene, and lower limb amputations are potential complications, with over half of all DFU cases becoming infected [5]. Having a DFU is a common precursor for amputation, with 85% of lower limb amputations preceded by foot ulceration [6]. The poor prognosis for DFUs emphasises the importance for health professionals to have a thorough understanding of the strategies to successfully manage DFUs to reduce DFU complications for people living with DFUs.

Long term management of a chronic disease such as diabetes mellitus can have widespread effects on individuals, including a negative outlook on life and feelings of sadness, worry, and despair [7]. Diabetes introduces a burden on the physical, psychological, and social contributors of quality of life in people living with the disease [8]. To the best of our knowledge, current literature investigating how people experience podiatric DFU treatment within chronic diabetes management is limited. There are currently no studies focusing on people’s experiences of podiatric treatment for DFUs to assist clinicians to improve the DFU care they provide to minimise the lifestyle and quality of life burden of DFU treatment and management procedures.

Effective treatment of a DFU requires a multi-faceted, multi-disciplinary approach involving healthcare professionals and the person with diabetes [9]. Management of DFUs involves prevention of infection, wound care, and metabolic control [10]. Offloading, in which load is redistributed from high pressure regions on the foot using non-removable casting or removable cast walkers are an important aspect of DFU treatment [10]. Podiatrists play a fundamental role in wound management and patient education focusing on foot selfcare habits for DFU prevention. However, a person’s ability to regularly use the prescribed equipment is a common challenge for podiatrists and other health professionals [11]. Previous studies including a cross-sectional analysis of 1743 patients with HIV suggested that patient-centred care incorporating patient-practitioner rapport can improve treatment compliance for various health conditions [12, 13]. It is important for clinicians to understand the challenges faced by people using prescribed DFU equipment to minimise the burden of these devices.

**Fig. 1:**
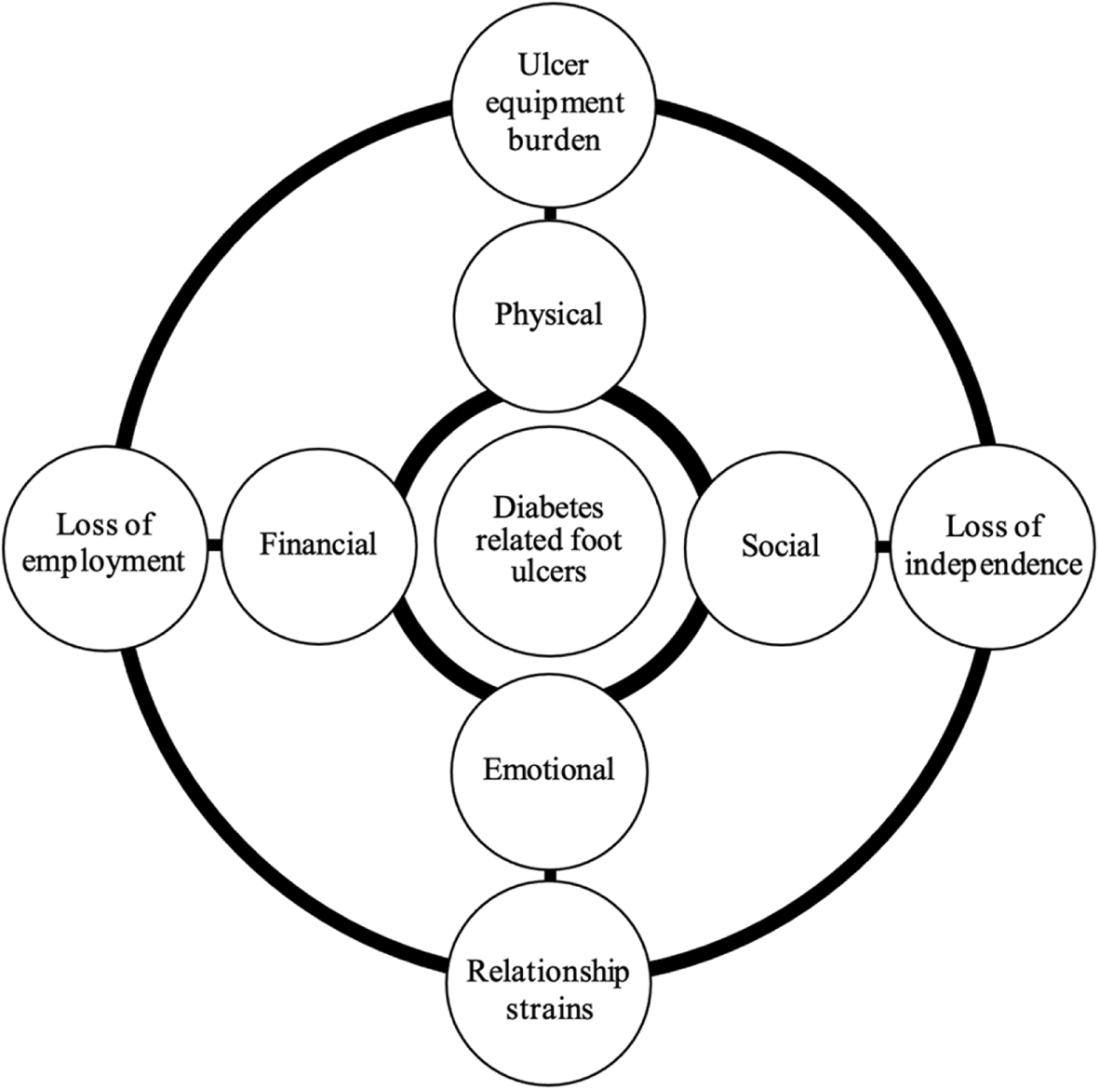
Multi-faceted impact of diabetes related foot ulcers on patients [9, 14]

Multi-disciplinary treatment and management of DFUs can have a broad impact on the physical, social, and emotional wellbeing of the individual (Fig. 1). Limitations in lifestyle activities that provided meaning for the person such as leisure or activities of daily living due to DFU management are well reported in qualitative studies to have negative psychological effects [9, 14, 15]. Yet how this relates to the podiatric treatment which people receive for DFUs remains unclear. Offloading management and time commitments to medical appointments for DFU treatment present significant restrictions in employment for many patients [16]. A study using focus group interviews of patients attending a diabetic foot clinic for DFU treatment found that DFU management introduced financial hardships and loss of stimulation previously provided from work [17]. Travel to medical appointments, parking fees, and costs of prescription devices are reported to contribute to the economic burden associated with DFU treatment [9, 18]. Foster and Lauver [19] conducted semi-structured interviews of 15 people with DFUs and identified financial burden and future uncertainty as predominant themes in their lives. Despite this, few studies describe the role of podiatric DFU care within this reported burden which limits the capacity of podiatrists to improve care experience for this group of people. This highlights a gap in the current body of professional knowledge. A qualitative descriptive interview study is a useful approach to better understand a person’s experience of a phenomenon such as podiatric DFU care.

Qualitative descriptive research enables readers to gain an understanding of a phenomenon from the perspective of the people experiencing it [20]. Semi-structured interviews in qualitative descriptive studies encourage participants to share descriptive recounts of their experience, to provide rich detailed data about the topic of interest [20]. A thorough understanding of the healthcare experience is essential to develop the most effective patient-centred treatment plan [17] and so qualitative research can inform health professional practice.

Therefore, this study aimed to:

1. Describe the experience of receiving podiatric care for DFUs in a tertiary care outpatient setting.
2. Identify key factors in podiatric care that influence this experience.

## Methods

This study is reported in accordance with the Consolidated Criteria for Reporting Qualitative Research (COREQ) checklist [21].

Ethical approval was obtained from the Central Adelaide Local Health Network (CALHN) and University of South Australia’s Human Research Ethics Committee (CALHN reference number 13567).

### Research design

A qualitative descriptive research design with thematic analysis was used to understand the experience of people engaging in podiatric care for a DFU [20]. Semi-structured interviews were used to gain a firsthand insight into the experience of podiatric care from people engaging with these services.

### Recruitment

Participants were purposively recruited from an outpatient podiatry clinic at a tertiary care hospital in the metropolitan region of South Australia to ensure they had experience of receiving podiatric care for a DFU in a tertiary care setting. Ten participants were recruited to provide sufficient data, as a minimum of six interviews has previously been described as sufficient in qualitative studies to develop an understanding of meaningful themes [22]. Participants who met the inclusion criteria (Table 1) were identified by podiatrists working at the clinic. No relationship was established between the participant and the principal researcher (EO) prior to the interviews.

**Table 1:** Inclusion/ exclusion criteria for participant selection

Inclusion criteria	Exclusion criteria
•18 + years old •Currently has a DFU •Is receiving treatment for a DFU in a podiatry department in a tertiary care health service (hospital)	•Currently has an ulcer/ history of ulcers of other aetiology (i.e., gout, cancer, rheumatoid arthritis or other non-diabetic disease) •Cognitive or communication disorder impairing ability to describe experience •Limited English proficiency impairing ability to describe experience in English

### Data Collection

An interview time was negotiated after participants provided written and informed consent (Additional file 1). Face-to-face interviews were conducted with the principal researcher (EO) in a meeting room at the clinic and each participant attended one interview. The principal researcher is a podiatry Honours student and was guided by three research supervisors with clinical and research experience in podiatry and physiotherapy. Three authors (CF, KG, RC) are health professionals with experience in conducting qualitative research with people engaged in healthcare and with health professionals and have trained and published in qualitative research. The principal researcher participated in interview training with the research team prior to data collection which included a mock interview by the principal researcher to pilot the interview guide and gain feedback on interview technique. A semi-structured interview guide was developed in conjunction with the research team, informed by the study topic, and similar studies investigating a person’s lived experience of podiatric care (Additional file 2) [23]. Primary open-ended questions and prompts to encourage rich descriptions of the participant experience were included in the interview guide. The semi-structured nature of the interviews allowed for new questions to be developed in response to participants’ stories. Demographic data was collected during the interviews. Interviews were audio recorded using a digital voice recorder. Interviews lasted between 10 and 26 min (mean 18 min). The principal researcher noted each participant’s observations and thoughts about their experience in a reflective journal to contribute to interpretation during data analysis. Interviews were transcribed using Descript software (Descript, San Francisco, CA, USA) and checked against the audio recording.

### Data analysis

Stepwise replication enhanced the dependability of the analysis method by ensuring all codes and themes were supported by participant data [22]. Thematic analysis was conducted using case themes as described by Peat and colleagues [24]. Initially, one transcript was chosen to be read separately by the principal researcher (EO) and all three researchers to immerse themselves in the data of a single participant. Initial comments and observations (codes) relating to the research question, ‘what is the experience of receiving podiatry care for DFUs?’, were identified by the researchers independently [24]. The primary researcher then met to discuss the first participant analysis with the other researchers. In these discussions, codes were compared and codes presenting similar ideas were grouped to form case themes [24]. Case themes for the single participant were discussed between researchers until agreed upon then summaries describing the salient points of the participant experience were written for each theme [25]. Each participant transcript was then analysed without comparison to the case themes from other participants’ interviews to support focussed interpretation of each lived experience [24]. At least two researchers analysed each transcript. When the 10 transcripts had been analysed, case themes from each transcript including summaries and coding for each theme were read, compared, and discussed by all the researchers. Each case theme was written on a Post-it™ note and visually displayed for consideration. The researchers collaboratively identified case themes that shared a similar meaning and could be grouped together into themes across the data set [25]. Following a reiterative process, four final themes were decided by the researchers to provide a description of the experience data as vividly as possible (Table 2) [26]. A summary of the coding tree is displayed in Additional file 3. Direct quotations from the participants were incorporated in the reporting of results to represent the participant voice [24]. All coding was conducted manually on paper due to limited access to coding software. After coding discussions between the research team, the principal researcher recorded the agreed coding in NVivo 12 Software (QSR International, Melbourne, Victoria, Australia) to generate coding reports that were shared between the research team and assisted final collaborative coding of case themes into four final key themes.

**Table 2:** Example of quotes, codes, case themes, and final themes

Quote	Code	Case theme	Final theme
*“Listen to your podiatrist. Do what they tell you” (P04)*	Trusting expertise	Trusting the podiatrist’s expertise	Trusting the podiatrists with the right expertise
*"People treated me how I felt I’d like to be treated and I’m a quieter person than some patients you get" (P06)*	Individualising care to the patient and their needs	Need to know what is going on to trust	Personalised care
*“They seem to treat everything alright. Their manner, their interest, everything. Everything about it is just good” (P05)*	Satisfied with experience	Satisfied with podiatry service and trust	Happy with the service, but not always with prescribed care
*“It’s holding me back, but most of all it’s holding the hospital back. I’ve got to keep on coming here to get the damage repaired” (P10)*	Not a straightforward path	Podiatry care is a long journey	It’s a long journey

Credibility, transferability, dependability, and confirmability are strategies to achieve rigour in qualitative studies [27]. To maintain credibility, peer debriefing was conducted with the supervisory team throughout data collection to minimise researcher bias [22]. A reflexivity debrief session with all members of the research team was conducted prior to data collection to share each researcher’s experiences and beliefs about the lived experience of podiatric DFU care and discuss how their views may influence individual understanding of participant experiences during data collection and analysis [24]. During the analysis, the two researchers with clinical podiatry experience provided insight into the podiatry care processes reported by participants and the researcher with a physiotherapy background had a role in discussions to question the perspectives of team members to minimise clinician bias. Demographic details of participants and quotations of the patient experience have been provided to allow the reader to determine the transferability of study findings to their local DFU patient population [28]. Confirmability was established using an audit trail to clarify the data collection and analysis procedures [22]. These techniques aimed to privilege the participant voice within their podiatric DFU treatment to ensure the findings accurately reflect each participant’s lived experience [24].

## Results

Ten participants were recruited during December 2020. Demographic data of the participants are listed in Table 3 and Additional file 4. All participants were male, with a mean age of 69.4 years and standard deviation of 15.3 years. Seventy percent of participants were retired and 30% were unemployed. Seventy percent were living at home with a partner or family member. Eighty percent of participants had been receiving podiatric DFU management for over nine months, with 70% of participants reporting a history of DFU infection.

**Table 3:** Participant demographic data

Participant code	Age	Employment status	Living arrangements	Duration of DFU (months)	Duration of DFU treatment (months)	Prior DFU (number)
P01	76	Retired	With a partner	3–4	2–3	Yes, 1
P02	95	Retired	With a partner	9 +	9 +	Yes, 1
P03	49	Unemployed	Alone	8–9	9 +	Yes, 4 +
P04	59	Retired	With a partner	3–4	3–4	No
P05	74	Retired	With a carer	9 +	9 +	No
P06	84	Retired	Alone	9 +	9 +	No
P07	63	Retired	With a partner	9 +	9 +	No
P08	51	Unemployed	With family	9 +	9 +	Yes, 2
P09	83	Retired	With a partner	9 +	9 +	Yes, 3
P10	60	Unemployed	With family	9 +	9 +	No

Four themes were identified to describe participants’ experience of podiatric care for DFUs: Trusting podiatrists with the right expertise; Personalised care; Happy with service but not prescribed equipment, and; It’s a long journey. Key factors in podiatric care that influenced participants’ experience were identified within the themes, including: demonstration of expertise, consistency in client-practitioner relationship, client-centred care, and innovation in equipment prescription.

### Theme 1. Trusting the podiatrists with the right expertise

Participants described the importance of trusting the podiatrist providing their DFU care as they perceived their DFU recovery to rely on the podiatrist’s expertise. Trust was associated with the podiatrist demonstrating professionalism in communication and behaviour such as, organisation and structure during appointments, and skill in DFU debridement and dressings. Use of good hygiene and infection control practices during DFU treatment also supported the participants’ trust of the podiatrist. Some participants specifically associated these qualities of expertise with the tertiary care context of the clinic. Trust helped participants to be confident in following the podiatrist’s advice and instructions about foot care,

“They’re the experts, so take their advice as much as possible and just make sure you do the right things” (P03).

“They know what they’re doing, they know more than me. I don’t know what they are doing anyways” (P02).

In contrast, participants lost trust when a podiatrist was perceived to not demonstrate the knowledge and skills expected of an expert practitioner. This included descriptions of “cutting too deep” during debridement or evidence of poor infection control and hygiene in the clinic such as inappropriate use of personal protective equipment.

“The only problem I had was a podiatrist over on the other side. He didn't seem to worry about hygiene or anything” (P02).

Three participants believed that a podiatrist’s debridement technique was the cause of further DFUs. One participant associated a change of dressing by a podiatrist with an infection in their DFU,

“If I hadn’t gone to the podiatrist in the first place, I might never have had an ulcer” (P01).

Building a relationship with the same podiatrist helped to build participants’ trust that the podiatrist was familiar with their DFU, and had the knowledge and skills to provide the most effective DFU treatment for them,

"It makes a big difference to who you know, and how you get on with them" (P06).

Many participants expressed they were hesitant to change podiatrists as they were concerned it would have a negative effect on their DFU management. One participant associated a negative DFU outcome with a change from the tertiary health clinic to a community podiatrist,

“They discharged me and sent me up the road, where I see a separate podiatrist. Like an outsource podiatrist. But then obviously the problem came back” (P03).

Despite their concern, participants perceived they had little control over the consistency of their treating podiatrist in the tertiary health setting due to the busy schedule of the clinic or the turnover of staff,

“Every time I get a good one, they get transferred” (P02).

This first theme describes the importance of perceived expertise for participants to have trust in podiatry care. Podiatrists strengthened trust through demonstrations of professionalism and expertise in DFU management. Consistency in the relationship between client and podiatrist was a key influence on trust.

### Theme 2. Personalised care

Throughout their experiences, participants described their appreciation for podiatric care being personalised to their needs and concerns. Three participants were particularly appreciative of the innovation shown by podiatrists in developing treatment plans to suit their personal goals and minimise interruptions to their lifestyle,

"She (podiatrist) is trying everything. If it doesn't work, it’s certainly not her fault or the hospital’s fault" (P07).

Participants wanted information from podiatrists about their DFU and care that was understandable to them and meaningful to their individual concerns and lifestyle. For some it was important to know about their DFU aetiology and six participants described a clear understanding of why they had an DFU,

“The first thing I would say to them, tell the person how they got the ulcer” (P01).

Other participants described the importance of knowing the relationship between the DFU and blood supply, with the risk of amputation recognised as a potential consequence of a poorly managed DFU,

“You know with diabetics, it’s not one problem. It’s eyes, feet, ulcers, blood pressure. The heart as well” (P03).

Four participants described not receiving the information they wanted from the podiatrist or the information was provided in a way they did not understand. These participants expressed disappointment and confusion about their lack of knowledge, yet perceived the responsibility for providing the information to be with the podiatrist,

“I was just stumped. It didn’t make sense to me. That something was going on with my feet” (P06).

“The people, they communicate, but they’re not telling me what I want to know. I’m not here to grill them” (P01).

This second theme describes the importance of personalised and client-centred care in participants’ experience of podiatry care for DFU. This was achieved by podiatrists collaborating with the client about their goals and treatment options. Effective provision of information addressed the person’s individual concerns about their condition and was provided in a way understandable to the individual.

### Theme 3. Happy with the service, but not always with prescribed care

Overall, participants were satisfied with the delivery of podiatric treatment they had received in the tertiary health clinic for their DFU but were burdened by the equipment prescribed and self-management expected of them between appointments. Visiting the podiatry clinic at the hospital was generally perceived as routine, comfortable, and uneventful,

“It’s fairly stress-free. It was easy to get in and out. Not too much drama” (P04).

Social interaction could be a valued part of the podiatric care experience, either with the podiatrist and other clinic staff, or with other people they met while travelling out of home for the appointment,

“It’s an outing, I’m usually home anyway. I go and have a coffee with some friends there every morning just about, then I’m home for the rest of the day” (P02).

In contrast to the service experience, the prescription of an offloading footwear device was consistently perceived to be burdensome. Most participants who had been prescribed equipment such as a cast walker described the rationale for offloading to allow the DFU to heal. They also acknowledged their responsibilities in managing the equipment between podiatry appointments,

“The secret to success is wearing the moonboot” (P07).

Yet they also reported use of the equipment to be a significant challenge and inconvenience to their lifestyle. These challenges experienced with adapting prescribed equipment into their lifestyle were perceived to not always be understood by the treating podiatrist. One participant described wearing his prescribed socks only when attending clinic appointments as he perceived his frustrations and inconvenience with the socks was not accepted by the podiatrist,

“They (podiatrist) didn’t like the idea that I wasn't wearing any socks. So, I wear socks when I come in here. I don't wear them normally” (P01).

In contrast, a few participants reported positive experiences when podiatrists recognised the difficulties and demonstrated innovation to adapt the prescribed equipment so their lifestyle burden was reduced,

“Last time she put in extra blocks in the orthotics and now she’s put a carbon plate in, she’s trying her best” (P07).

Burdens of offloading devices were experienced as both physical and psychological consequences, with some participants complaining of physical exhaustion from wearing a removable cast walker every day. The challenges experienced by participants with DFU offloading equipment and dressings forced some into a more sedentary lifestyle,

“You walk around for a long time but it’s like walking with a concrete brick” (P03).

Social roles and meaningful activities were negatively impacted by commitments to DFU management procedures. These included difficulties caring for grandchildren and attending school drop-off when using a removeable cast walker, and difficulties taking the cast walker on and off limiting the participant’s ability to drive a car to meet friends or attend a workplace,

*"It's frustrating not being able to just get in the car and go where I feel like going, makes a difference and friends have to come and see me rather than me going to see them” (P09).*

Keeping DFU dressings dry and clean was also described as a problem for many participants which prevented participation in outdoor leisure activities including going to the beach, riding a bike or going on walks,

"I can't go to the water. I haven't been in the pool for two years" (P09).

This third theme describes the widespread challenge and burden of using prescribed equipment during podiatry care for DFU. This burden often had negative effects on a participants’ ability to participate in meaningful roles and activities. The negative experience of equipment use was not always recognised by the podiatrist. The experience of using equipment could be improved by podiatrists innovating use of equipment to better meet the participant’s needs.

### Theme 4. It’s a long journey

Podiatric care for a DFU was experienced by all participants as a long and interrupted journey. Six participants struggled with acceptance of their DFU and implementing offloading equipment into their lifestyle. Several participants had almost healed their DFU before experiencing an DFU infection and the need for continued treatment,

"And then it (ulcer) breaks down again. It's a never-ending cycle" (P10).

“It’s one step forward and two steps back” (P05).

Participants recognised that despite engaging in regular podiatric DFU treatment, the healing process for a DFU could not be rushed.

*"It's going to take a long time. It's just something that you can't rush. If you rush it, you'll get to a certain point and just break down again. Then you’re back to square one" (P10).*

Participants were grateful for emotional support and medical attention they received from podiatrists and other health professionals during this long journey,

“There were vascular surgeons, three or four nurses, I felt like a real king” (P07).

However, the journey of podiatric DFU treatment included unpleasant experiences. Infection of a DFU was a common and significant setback for several participants which created doubt about timely DFU resolution. The need to return to work presented setbacks for one participant as the physical requirements of their work delayed DFU healing. The long, interrupted duration of podiatric DFU care had significant impacts on the wellbeing of participants,

"Your legs are your pillars. So, having any wounds affect your mobility or your way of life is life changing" (P08).

Countless setbacks when engaging with podiatric care for their DFU was associated with some participants demonstrating a pessimistic outlook towards timely DFU resolution and their return to a regular lifestyle. Guilt and frustration were also experienced by participants whose partners were required to increase household workload and transport them to and from podiatry appointments. Two participants experienced difficulty maintaining full time employment due to time commitments attending frequent podiatry appointments and use of a removeable cast walker.

For most participants, the DFU was a co-morbidity to other chronic health problems. Sometimes the DFU was experienced as less of a concern compared to significant medical conditions,

“When you've had your chest cut open, this is nothing. It's a fairly minor inconvenience” (P04).

However, many participants experienced significant stress managing podiatric DFU treatment alongside their co-morbidities,

“Having a medical condition on top is like having the weight of the world on your shoulders” (P08).

This final theme describes the long and frequently interrupted duration of podiatry care for participants. The common experience of infections and slow healing had significant effect on lifestyles and wellbeing can be acknowledged and support offered by podiatrists during delivery of care.

## Discussion

This study aimed to investigate how people with DFUs experience podiatric DFU care by gaining an insight from the people engaging in podiatry care through a qualitative interview technique. A person’s ability to trust their podiatrist was a key facilitator for a positive treatment experience. Trust was achieved when podiatrists demonstrated professional expertise in communication, DFU debridement, and dressings. This is consistent with reported experiences of receiving treatment for rheumatoid arthritis-related foot ulcers [29]. Participants reflected on previous experiences when trust for a podiatrist was lost due to perceived poor hygiene standards or demonstration of knowledge. As a result of this loss of trust, participants demonstrated doubt towards the value of podiatry for DFU management and the role of regular podiatry treatment for DFU prevention which threatens engagement with necessary healthcare. This finding builds on previous research which has identified trust for health professionals correlates with higher patient satisfaction [30].

Consistency in podiatrist and treatment is another factor that helped people to trust the quality of their podiatry care. A person’s familiarity with their health professional has been shown to help establish therapeutic patient-professional relationships in previous studies [29]. This emphasises the need for podiatry clinics to offer consistency of clinician to a person with a DFU. If change of podiatrist is indicated, an explanation describing the rationale for this change in relation to the person’s individual DFU circumstance and confirming the expertise of the new podiatrist or offering a choice of podiatrist could reduce patient distrust.

The study’s findings suggest that podiatrists need to provide individualised information to people with DFUs for the information to be useful to the person. This is supported by Calnan and Rowe [31] who highlighted the importance of tailoring health advice to meet patient needs and establish trust. Participants’ desires for meaningful information in this study is consistent with the expectations of information in studies of experiences of other foot-related conditions. A study of individuals with plantar heel pain indicated they wanted clear explanations about their condition’s aetiology and treatment [32]. However, this desire for information was not coherent amongst all participants in this study with some expressing indifference to information provided by podiatrists. This highlights that personalising information includes tailoring it to both the individual’s expectations and their health literacy to facilitate positive outcomes. The study findings support open communication facilitated by a trusted relationship can enable podiatrists to gain an insight into an individual’s challenges to tailor information and interventions to their needs.

Prescribed equipment including removable cast walkers and dressings were a significant burden in daily activities for many participants. Previous studies have described the lifestyle limitations presented by DFU offloading devices [33]. Ulcer equipment was a burden to family and carer roles for some participants. This is supported by Kinmond et al. [34] who reported restrictions in activity participation and relationships as a result of daily use of DFU equipment. These lifestyle limitations can affect an individual’s quality of life and psychological wellbeing living with a DFU [34]. This study found that even when participants had trust in their podiatrist and understood instructions about DFU care, the limitations of the equipment could make it too difficult for them to use daily. Podiatrists can support a person’s capacity to effectively use prescribed equipment by remaining abreast of new technology and best available evidence, and using this knowledge to individualise treatment by adapting or trialling equipment to reduce its limitations on meaningful activities.

Setbacks in DFU recovery during podiatry care were experienced by participants as negative impacts on employment, personal independence, and return to meaningful activities. This is consistent with current literature describing lifestyle burden from DFU management including a sense of guilt due to an increased dependence on others for daily needs [3, 9]. Our results indicated that the use of dressings and offloading devices significantly contribute to lifestyle burden. Moreover, commitments to podiatric DFU treatment between appointments limited opportunities for full time employment as participants felt pressured to keep their DFU clean, dry, and protected to promote timely healing and felt unable to manage this in a workplace. Although no participants expressed a financial burden due to this occupational limitation, which has been previously described in the literature [9, 18], this may have been because most of the sample were retired.

Living with a chronic illness such as diabetes mellitus can introduce substantial psychological impacts as people with the chronic illness can struggle with use of the prescribed treatment [35]. Consistent with previous research involving people with diabetes mellitus, many participants in this study struggled with acceptance of their DFU and the timeline and burden of its associated podiatric treatment [35]. Participants demonstrated a pessimistic outlook for their foot health which has also been identified in other studies [29]. To support the psychological health of people receiving treatment for a DFU, it is important for health professionals to empathise with the person and attempt to understand the lifestyle impacts of DFU treatment in order to tailor treatment strategies to minimise burden and promote positive patient experiences.

### Limitations and further research

Limitations of this study must be considered before implementing the findings into clinical practice. Information about the participant’s DFU including infection, duration of DFU and podiatric treatment was self-reported, no medical records were accessed, and the researchers did not assess the participant’s DFU. Semi-structured interviews provide participants with one opportunity to report their lived experiences and therefore, they may not provide a full report of their experience if they do not recall the details at the time of the interview.

A sampling bias may have influenced the lived experiences described in this study. All participants were receiving public sector healthcare and many participants were referred from other hospitals within the same local health network. Therefore, these experiences may not be reflective of people receiving private healthcare only or people living in other locations. Future research to understand the lived experience of podiatry care for people with DFUs could compare experiences between public and private healthcare or investigate experiences of podiatric DFU treatment across metropolitan and rural South Australia.

All participants in this study were male as most people receiving DFU treatment in that podiatry department were male. People who associate with other genders may have other values which influence their experience of podiatric DFU management including familial roles and expectations with use of DFU equipment. Additionally, all participants were not employed at the time of the study. Further research can improve our understanding of the impacts of podiatric DFU treatment on occupation.

## Conclusion

This study found that podiatric DFU treatment is a lengthy and onerous journey for many people with DFUs. The podiatric management of DFUs with offloading equipment and dressings was shown to be a significant contributor to treatment burden and can have negative impacts on a person’s independence in social and daily activities. People with DFUs felt confident in their DFU treatment when they trusted their podiatrist. A trusting relationship was supported by consistency in the podiatrist who provided the DFU treatment and demonstration of high organisational and hygiene standards. A podiatrist’s willingness to personalise information to address an individual’s concerns and be innovative in adaptation of DFU equipment to minimise the burden on a person’s roles and responsibilities can improve a person’s experiences with podiatric treatment for a DFU. Consideration by podiatrists of a person’s physical, occupational, and psychosocial lifestyle factors which impact their experience with podiatric DFU treatment will support development of a personalised and achievable treatment plan.

## Supplementary Information

**Additional file 1.** Consent Form.

**Additional file 2.** Interview questions.

**Additional file 3.** Coding Tree.

**Additional file 4.** Additional participant demographic data.

## Data Availability

Transcripts will not be made available to maintain confidentiality of the participants.
