# Supplementary material for: Investigating the experience of receiving podiatry care in a tertiary care hospital clinic for people with diabetes related foot ulcers
Source: J Foot Ankle Res. 2022 Jul 1;15:50. doi: 10.1186/s13047-022-00556-1 (PMC9248168; doi:10.1186/s13047-022-00556-1)
Supplement: Supplementary file 1 — Additional file 1. Consent Form. [file 13047_2022_556_MOESM1_ESM.docx]

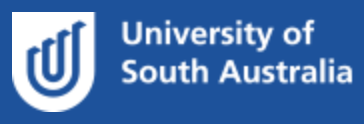

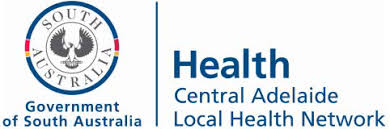


**Investigating the lived experience of people with diabetic foot ulcers**

CALHN Reference Number: 13567

PI Name: Emilee Ong

Location: The Queen Elizabeth Hospital, Royal Adelaide Hospital, and the University of South Australia City West/ City East Podiatry Clinic

- I have read the Participant Information Sheet or someone has read it to me in a language that I understand.
- I have had an opportunity to ask questions and I am satisfied with the answers I have received.
- The nature, purpose and risks of the research project have been explained to me. I understand them and agree to take part.
- I freely agree to participate in this research project as described and understand that I am free to withdraw at any time during the project without affecting my future healthcare.
- I understand that I will be given a signed copy of this document to keep.

Name of Participant (please print) ____________________________________________

Signature ___________________________________ Date ________________________

Name of Participant (please print) ______________________________________

Signature ____________________________ Date ________________________

Name of Participant (please print) ____________________________________________

Signature ___________________________________ Date ________________________

Name of Participant (please print) ______________________________________

Signature ____________________________ Date ________________________

**Declaration by Senior Researcher**

I have given a verbal explanation of the research project, its procedures and risks, and the implications of withdrawal from the research project and I believe that the participant has understood that explanation.

Name of Senior Researcher (please print) ______________________________________

Signature ___________________________________ Date ________________________

Name of Senior Researcher (please print) _______________________________

Signature ____________________________ Date ________________________

Name of Senior Researcher (please print) ______________________________________

Signature ___________________________________ Date ________________________

Name of Senior Researcher (please print) _______________________________

Signature ____________________________ Date ________________________
