# Supplementary material for: Investigating the experience of receiving podiatry care in a tertiary care hospital clinic for people with diabetes related foot ulcers
Source: J Foot Ankle Res. 2022 Jul 1;15:50. doi: 10.1186/s13047-022-00556-1 (PMC9248168; doi:10.1186/s13047-022-00556-1)
Supplement: Supplementary file 2 — Additional file 2. Interview questions. [file 13047_2022_556_MOESM2_ESM.docx]

**Interview questions**

Today, I am really interested in hearing about what it is like for you to have podiatry treatment for your ulcer. But first, I’d like to understand a little bit more about who you are…

**Demographic data**

Self

Please tell me a bit about yourself:

- What is your age and the gender you identify with?
- Where and when were you born and raised?
- Where are you living now? What is your postcode there?
- Are you living with a partner, someone else or living alone?
- Can you describe your occupation? Are you working at present?

Ulcer history

Please tell me a bit about your ulcer:

- How long have you had this ulcer for?
- How long have you been attending this podiatry clinic for ulcer treatment?
- Have you had a previous ulcer or is this your first foot ulcer?
- Can you describe any medical problems you have had with your ulcer (i.e. Infection)?
- Who referred you to this podiatry clinic?
- Are you seeing any other health professionals about your ulcer (diabetes educator, exercise physiologist, physiotherapist, GP etc)?

**Ulcer**

Probes to use with each story

- How did that make you feel? What helped you to manage those feelings?
- How did you make that decision (family, friends, doctor, podiatrist, internet) or what helped you to make that decision?

Specific treatment experiences

- Tell me about the last time you came to the clinic for treatment of your foot ulcer?
- Now can you tell me about the first time you came to the clinic for treatment of your foot ulcer?

Possible prompt example

- What has the change in podiatrist been like for you?

Memorable moments

- Can you describe a certain experience that you have had in the clinic that stands out in your mind?

If needed questions (e.g. if participant has not shared much data up to this point or you think you could gain more)

- Tell me about a good experience you have had in the podiatry clinic.
- Tell me about an experience in the podiatry clinic that has not been good for you, or that you think could have gone better.

Lifestyle changes

- Tell me about a time when having treatment for a foot ulcer changed your normal routine.

Future outlook

- What advice would you give to podiatrists who are treating people with foot ulcer?
- What advice would you give to a person who needs podiatry treatment for a foot ulcer?

End

- Is there anything else you would like to share with me about your experiences with podiatry treatment for your foot ulcer?
- Have we missed anything you think is important?
- Is there anything you would like to ask me?

**Probes**

- Please tell me more about that.
- Then what happened?
- Can you give me an example of what you mean?
- How does that make you feel?
- Please tell me more about that time.
- How has that changed over time?
