# Supplementary material for: Investigating the experience of receiving podiatry care in a tertiary care hospital clinic for people with diabetes related foot ulcers
Source: J Foot Ankle Res. 2022 Jul 1;15:50. doi: 10.1186/s13047-022-00556-1 (PMC9248168; doi:10.1186/s13047-022-00556-1)
Supplement: Supplementary file 3 — Additional file 3. Coding Tree. [file 13047_2022_556_MOESM3_ESM.docx]

**Table 4** Summary of codes which contributed to the four themes

| Trusting the podiatrists with the right expertise | |
| --- | --- |
| Trusting the podiatrist expertise | P03 |
| Trusting podiatrists’ expertise | P07 |
| Trusting expertise | P04 |
| Trusting professional expertise | P02 |
| They try everything to help me (innovative) | P07 |
| Unsure whether to trust podiatry (community vs private) | P01 |
| Different trust with different clinics (community vs private) | P03 |
| Good podiatrists practice good hygiene | P02 |
| Good vs bad cutting and dressing (community vs private) | P06 |
| Consistency in podiatrist is important | P10 |
| Consistency builds trust | P06 |
| Consistency builds podiatrist-patient rapport | P09 |
| Professionalism is consistency and attention | P07 |
| Timeliness is important in professionalism | P06 |
| Personalised care | |
| Have a good understanding about my ulcer | P03 |
| Want to stop another ulcer happening | P01 |
| Need to know what is going on to trust | P06 |
| No one has told me | P05 |
| They don’t tell me what I want to know | P01 |
| Happy with the service, but not always with prescribed care | |
| Happy with appointments and ulcer management | P03 |
| Podiatry is routine and comfortable | P09 |
| Happy with podiatry service | P04 |
| Satisfied with podiatry service and trust | P05 |
| It’s a social outing for me | P02 |
| Keeping my legs is important (treatment helps) | P08 |
| I do the right thing by following their advice | P02 |
| Using a moonboot and medical grade footwear is frustrating | P03 |
| The boot is a burden | P07 |
| It is an inconvenience and nuisance | P01 |
| Ulcer management restricts your normal lifestyle | P04 |
| It’s a long journey | |
| Accepting the ulcer and podiatry treatment | P08 |
| Podiatry is a long journey | P10 |
| Ulcer healing takes time | P07 |
| Challenges with ulcer management | P03 |
| Many setbacks | P05 |
| Physical environment is important for healing | P10 |
| Lifestyle impact of ulcer management | P04 |
| Ulcer management has negative employment and financial impacts | P10 |
| Having an ulcer is lifechanging | P08 |
| The good and bad of having an ulcer | P08 |
| Ulcer healing has ups and downs | P02 |
| Ulcer affects mobility | P05 |
| There are worse things than an ulcer | P04 |
