# Supplementary material for: Investigating the experience of receiving podiatry care in a tertiary care hospital clinic for people with diabetes related foot ulcers
Source: J Foot Ankle Res. 2022 Jul 1;15:50. doi: 10.1186/s13047-022-00556-1 (PMC9248168; doi:10.1186/s13047-022-00556-1)
Supplement: Supplementary file 4 — Additional file 4. Additional participant demographic data. [file 13047_2022_556_MOESM4_ESM.docx]

**Table 5** Additional participant demographic data

| Participant code | Ulcer symptoms | Referral to clinic | Other health professionals |
| --- | --- | --- | --- |
| P01 | Nil reported | Wife was receptionist at hospital podiatry clinic | GP |
| P02 | Infection | Multidisciplinary Foot Clinic at The Queen Elizabeth Hospital | Nurse |
| P03 | Infection | GP | GP, eye specialist, heart specialist, endocrinologist |
| P04 | Infection | Royal Adelaide Hospital | GP, eye specialist, heart specialist, kidney specialist |
| P05 | Nil reported | Hampstead Rehabilitation Centre | Nurse, physiotherapist |
| P06 | Nil reported | GP | GP |
| P07 | Infection | Endocrinologist | GP, stomach doctor, cardiologist, endocrinologist, neurologist |
| P08 | Infection | GP | GP, RDNS nurse |
| P09 | Infection | Vascular surgeon | GP, infectious disease doctor, vascular surgeon |
| P10 | Infection | GP | GP, orthopaedics |
